# Supplementary material for: Protocol for an interdisciplinary cross-sectional study investigating the social, biological and community-level drivers of antimicrobial resistance (AMR): Holistic Approach to Unravel Antibacterial Resistance in East Africa (HATUA)
Source: BMJ Open. 2021 Mar 8;11(3):e041418. doi: 10.1136/bmjopen-2020-041418 (PMC7942251; doi:10.1136/bmjopen-2020-041418)
Supplement: Supplementary data [file bmjopen-2020-041418supp001.pdf]

Appendix A. Data summary of HATUA Pilot data relating to patient recruitment

Table A1: Summary of socio-demographic characteristics of patients recruited in Mbarara district, Uganda.

	N	%
**Gender**		
Male	38	29.5
Female	91	70.5
Missing	0	0
**Age**		
Less than 18	14	10.9
18-34	57	44.2
35-54	47	36.4
55 and over	9	7.0
Missing	2	1.5
**Education**		
None	30	23.2
Primary	79	61.3
Secondary	14	10.9
Tertiary	4	3.1
Missing	2	1.5
**Taken medication in the past 6 months**		
No	38	29.5
Yes	72	55.8
Missing	19	14.7
**Of those who did, where obtained medication from (multiple choice possible)**		
Clinic/ health centre	67	93.0
Drug seller	13	18.0

Appendix B. Agreed set of clinically relevant ABs for AST

AB Class	Generic Name
Penicillins + β-lactamase inhibitors	Amoxicillin/clavulanate
Anti-staphylococcal β-lactams	Cefoxitin
ESBL Cephalosporins	Ceftazidime
ESBL Cephalosporins	Ceftriaxone
Fluoroquinolones	Ciprofloxacin
Macrolides	Erythromycin
Aminoglycosides	Gentamycin
Oxazolidinones	Linezolid
Quinolone	Nalidixic Acid
Nitrofuran	Nitrofurantoin
Folate pathway inhibitors	Sulfamethoxazole
Tetracycline	Tetracycline
Folate pathway inhibitors	Trimethoprim
Glycopeptides	Vancomycin
